# Therapeutic approaches for the treatment of new onset and flared juvenile systemic lupus erythematosus with active renal disease: an international multicenter PRINTO study

**DOI:** 10.1186/1546-0096-9-S1-P258

**Published:** 2011-09-14

**Authors:** P Miettunen, A Pistorio, A Ravelli, S Oliveira, M Alessio, R Cuttica, D Mihaylova, G Espada, S Pasic, E Cortis, S Ozen, O Porras, F Sztajnbok, E Palmisani, A Martini, N Ruperto

**Affiliations:** 1 IRCCS G. Gaslini, Pediatria II, Paediatric Rheumatology International Trials Organisation (PRINTO

## Objectives

To evaluate in a prospective international cohort of juvenile systemic lupus erythematosus (JSLE) with active renal disease (ARD) response to therapy over a 24-month period.

## Patients & Methods

134 new onset and 106 flared JSLE patients with ARD, age < 18 years, were included. JSLE disease activity parameters and therapeutic approaches were analyzed at baseline, 6, 12 and 24 months, in 4 geographic areas. Response was assessed according to the PRINTO/ACR JSLE criteria “as observed” and as “per intention-to-treat (ITT)”.

## Results

New patients had higher baseline disease activity compared to flared patients, but initiated corticosteroids at similar doses. Cyclophosphamide was the most common (41.2%; 99/240) immunosuppressive medication, followed by azathioprine (25%; 60/240). Mycophenolate mofetil and azathioprine were more commonly used in flared patients. Patients from Latin America received more pulses and higher doses of steroids when compared to Western Europe. The use of cyclophosphamide was similar in all 4 regions. In the “as observed” analysis 78% (103/132) of new compared to 57.4% (58/101) of flared JSLE patients (p=0.0007) reached at least PRINTO/ACR 70 level of response at 6 months, which increased to 87.1% in new and 77.2% in flared patients at month 24 (p=0.12). Corresponding figures for the ITT analysis were similar and 80% (68/85) of new vs 73.7% (42/57) of flared patients were able to maintain the initial assigned therapy over 24 months (p=0.38). Corticosteroids were discontinued in 28.6% (20/143).

## Conclusions

Both new and flared patients improved similarly over 24 months. Some differences in therapeutic approaches exist worldwide.

